# Identification of a Metagenome-Assembled Genome of an Uncultured *Methyloceanibacter* sp. Strain Acquired from an Activated Sludge System Used for Landfill Leachate Treatment

**DOI:** 10.1128/MRA.00771-20

**Published:** 2020-08-06

**Authors:** Shohei Yasuda, Toshikazu Suenaga, Laura Orschler, Shelesh Agrawal, Susanne Lackner, Akihiko Terada

**Affiliations:** a Department of Chemical Engineering, Tokyo University of Agriculture and Technology, Koganei, Tokyo, Japan; b Global Innovation Research Institute, Tokyo University of Agriculture and Technology, Fuchu, Tokyo, Japan; c Department of Civil and Environmental Engineering Science, Institute IWAR, Chair of Wastewater Engineering, Technical University of Darmstadt, Darmstadt, Germany; University of Maryland School of Medicine

## Abstract

Using metagenome sequencing, a nearly complete genome sequence was retrieved for the uncultured *Methyloceanibacter* sp. strain A49, recovered from an activated sludge system used for landfill leachate treatment at a closed landfill site. The total size and encoded sequences are 3,407,434 bp and 3,280 genes, respectively.

## ANNOUNCEMENT

Until now, methylotrophic bacterial species of the genus *Methyloceanibacter*, belonging to the order *Rhizobiales* (class *Alphaproteobacteria*), have been discovered only in marine environments (1–3). Here, we report for the first time the metagenome-assembled genome (MAG) sequence of an uncultured *Methyloceanibacter* species retrieved from the activated sludge tank of a landfill site that was closed for nitrogen removal from the leachate.

An activated sludge sample was taken from an intermittently aerated sequencing batch reactor removing nitrogen from landfill leachate at a closed landfill site (Tokyo, Japan). The reactor received a periodic methanol supply as an external electron donor, and the leachate had a temperature, pH, total nitrogen concentration, and chloride ion concentration of 28.3°C, 7.65, 159 mg N/liter, and 7,693 mg/liter, respectively. DNA was extracted using the Fast DNA spin kit for soil (MP Biomedicals, Santa Ana, CA, USA) according to the manufacturer’s protocol. The quality of the DNA was checked using gel electrophoresis, and the concentration was measured using a Qubit 3.0 fluorometer (Thermo Fisher Scientific, Waltham, MA, USA). Enzymatic shearing was performed with the Ion Xpress Plus fragment library kit (Thermo Fisher Scientific) to prepare fragmented libraries from genomic DNA for sequencing. Size selection of the library for 600-bp reads was performed with E-Gel Size Select II agarose gel, and libraries were tagged using Ion Xpress barcode adapters (Thermo Fisher Scientific). Template preparation was performed on an Ion Chef system with the Ion 520 and Ion 530 ExT kit. Sequencing was performed on the Ion Torrent Ion S5 system using the 530 chip. Torrent Suite v. 4.4.2 (Thermo Fisher Scientific GmbH, Dreieich, Germany) was utilized for base calling with default parameters according to the manufacturer’s protocol, which resulted in 20,244,817 single-end reads with a mean length of 334 bp. Quality filtering of the metagenomic reads was performed using Trimmomatic v. 0.36 (4). Trimming was applied to both sides of the reads to ensure a Q20 quality score, while maintaining a minimum read length of 250 bp. After trimming, 13,005,150 total reads were assembled using MEGAHIT v. 1.1.3 (5) at the minimum contig length of 1,000 bp, producing 44,392 contigs (total, 104,856,336 bp; *N*_50_, 8,139 bp; and *L*_50_, 2,909 bp). Bowtie 2 v. 2.3.4.2 (6) and SAMtools v. 1.9 (7) were used for mapping the reads. After converting the sequence alignment map (SAM) files to binary alignment map (BAM) files with SAMtools v. 1.9 (7), single-copy bacterial and archaeal genes were identified using HMMER v. 3.2 (8). Default parameters were used for all software unless otherwise specified.

Unsupervised binning by CONCOCT v. 0.4.0 (9) and subsequent manual curation by Anvi’o v. 5 (10) were used to obtain MAGs (with more than 70% completeness and less than 10% redundancy) from the metagenomic assembly. The taxonomic classification of the MAGs was verified using reference genomes from NCBI RefSeq with MiGA (http://microbial-genomes.org) (11). One of the MAGs retrieved by MiGA is proximal to *Methyloceanibacter* sp. strain wino2 (GenBank accession number NZ_CP028960), and the average nucleotide identity (ANI) is 81.38%, suggesting that the MAG may represent a novel *Methyloceanibacter* species. The completeness and contamination are 91.39% and 1.68%, respectively, using CheckM v. 1.0.7 with taxonomic-specific workflow (phylum *Proteobacteria*) (12).

The genome of A49 was annotated using the DDBJ Fast Annotation and Submission Tool (DFAST) pipeline (13). The genome length is 3,407,434 bp, it consists of 447 contigs, and it has a GC content of 62.73%, 26× average coverage, an *N*_50_ value of 10,298 bp, 1 rRNA, and 42 tRNAs. Functional annotation with KofamKOALA (14) revealed that the MAG harbors the *xoxF* gene, which encodes a pyrroloquinoline quinone-dependent methanol dehydrogenase (15), suggesting that the MAG has a possible methanol oxidation function.

### Data availability.

The MAG sequence of *Methyloceanibacter* sp. strain A49 has been deposited in DDBJ/EMBL/GenBank under the accession numbers BLYM01000001 through BLYM01000447. The BioSample accession number is SAMD00231436. The read data were also deposited in DDBJ Sequence Read Archive (SRA) under the SRA experiment accession number DRX196218 (run number DRR205819).
